# Application of chromatographic techniques in the analysis of total nitrosamines in water

**DOI:** 10.1016/j.heliyon.2020.e03447

**Published:** 2020-03-03

**Authors:** Abdulrazaq Yahaya, Damilola Babatunde, Lamidi W.B. Olaniyan, Oluranti Agboola

**Affiliations:** a Department of Chemistry, Kogi State University, Anyigba, Kogi State, Nigeria; b Department of Environmental, Water and Earth Science, Faculty of Science, Arcadia Campus, Tshwane University of Technology, Pretoria, South Africa; c Department of Chemical Engineering, Covenant University, Ota, Nigeria; d Biochemistry Department, Faculty of Basic Medical Sciences, Ladoke Akintola University of Technology, Ogbomoso, Nigeria

**Keywords:** Analytical chemistry, Electrochemistry, Metals, Nitric oxide, n-nitrosodimethylamine, Column, Carcinogenic, Chromatography

## Abstract

The use of ozone, chloramine and chlorine dioxide for water treatment results in the formation N-nitrosamines in the treated water. These groups of chemicals and other nitrogen-containing compounds have been described as disinfection by-products (DBPs) which are known for their toxicity. Nitrosamines are a potential source of nitric oxide (NO) which can bind with metals present in the sample matrix leading to formation of metal – nitrosyl complexes and dissolved metals have the potential to increase the total nitrosamines in water. This phenomenon has not received the desired attention and determination of metal-nitrosyl complexes lack standard analytical technique. Chromatography linked to various detectors is the commonest of the techniques for nitrosamine analysis but it is beset with reduced sensitivity as a result of inappropriate choice of the column. Incidentally, chromatographic techniques have not been really adapted for the analysis of metal-nitrosyl complexes. Therefore, there is need for the survey of existing techniques vis-à-vis metal-nitrosamine analysis and to suggest possible areas for method optimization.

## 1 Introduction

The occurrence of nitrosamines (NAms) as emerging disinfection by-products (EDBPs) in drinking water has been ascribed to the use of chloramine, ozone and chlorine dioxide as chemical oxidants for water treatment [1, 2]. The formation of these contaminants stems from the interaction between the residual disinfectants and the organic matters present in the water [3, 4, 5]. The ubiquity of NAms has received the attention of various authors recently [6, 7, 8]. Jurado-Sánchez et al. [9] reported 18 ng/L of total nitrosamines in drinking water treatment plant in Spain. The presence of these carcinogens has also been reported in various water sources from other parts of the world (Table 1).

**Table 1:** Nitrosamine contaminants in water.

Nitrosamine	Concentration (ng/L)	Water source	References
Total nitrosamines	60.8	Songhua River, China	[10]
NDMA	4.3 2.2	Raw and finished water, Japan.	[11]
NDMA	180	Water treatment plant, Canada	[12, 13]
NDMA	<2	Wastewater, USA	[14]

The volatile (N-nitrosodimethylamine; NDMA, N-nitrosodiethylamine; NDEA and non-volatile, (N-nitrosoproline; NPRO; N-nitrososarcosine (NSAR) nitrosamines and nitrogen containing disinfection by-products (DBPs) are known to be more toxic than the regulated DBPs [3, 15, 16]. Nitrosamines such as N-nitrosodimethylamine (NDMA) are carcinogenic in rat liver [10, 17]. The presence of nitrosamines in wastewater, source water and drinking water is an emerging issue and of health concern [3, 18]. The United State Environmental Protection Agency (US EPA) added nitrosamines to unregulated organic pollutants and as “probably carcinogenic” (Table 2) [10, 14, 19, 20]. Different permissible levels have emerged for nitrosamines in different countries. For example, 10 ng/L was the maximum permissible limit in California [21] and in Germany. Whereas in Netherland 12 ng/L was the contaminant limits for NDMA in drinking water while Ontario, accepts the maximum limit of 9 ng/L for NDMA [13, 16].

**Table 2:** List of carcinogenic N-Nitrosamines [20, 22, 23].

Nitrosamines	Formula	US EPA MCL (ng/L)	Log Kow	Water Solubility mg∖L	US EPA Cancer classification.	Risk Level (ng/L) × 10^−6^
NDMA	C_2_H_6_N_2_O	7	- 0.57	1,000,000	B2	3.0
NMEA	C_3_H_8_N_2_O	20	0.04	300,00	B2	1.5
NDEA	C_4_H_10_N_2_O	2	0.48	106,000	B2	1.0
NDPA	C_6_H_14_N_2_O	50	1.36	13,000	B2	5.0
NDBA	C_8_H_18_N_2_O	60	2.63	1,270	B2	3.0
NPYR	C_4_H_8_N_2_O	200	- 0.19	1,000,000	2B (IARC)	15.0
NPIP	C_5_H_10_N_2_O	na	0.36	76,480	B2	3.5
NDPHA	C_12_H_10_N_2_O	70,000	3.13	35	B2	na
NMOR	C_4_H_8_N_2_O_2_	na	- 0.44	861,527.5	2B (IARC)	na

IARC: International Agency for Research on Cancer; NA: Not available, MCL: Maximum contaminant level.

The nitrosyl group present on nitrosamines behave as electron donor (NO+), electron acceptor (NO-) as well as radical (NO*) resulting into formation of metal complexes.

The reactivity of NO radicals ensures interaction with metals present in the sample forming nitrosamine-metal complex [24, 25, 26, 27]. The analytical significance of this interaction has not been properly investigated. A survey of methodologies available for the analysis of nitrosamines in water shows that the chromatographic techniques are in the forefront of others (Table 3). The widely used method specifically is gas chromatography linked to mass spectrometric detector with carefully selected column.

**Table 3:** Chromatographic methods for determination of nitrosamines.

Matrix	Compounds	Sample Preparation	Analytical Instruments	Analytical Column	Detector	LOD	LOQ	RSD (%)	Recovery (%)	Reference
River Water	NDMA, NDEA, NDPA, NMEA, NDBA, NDPHA NDMA–d6	SPE (CCC) Restek (cat. #26032). USEPA Method 521	GC Agilent 6890 N	Rtx 5SiL MS (30 m × 0.25 mm ID × 1.0 μm).	Agilent MS 5973.	2.5–40.6 ng/L	7.9–127.7 ng/L	<15	72.3–98.6	[54]
Potable water	NDMA-d6NDMA, NDPA, NPIP, NMEA, NDEA, NPYR, NDBA,	SPE (CCC) Restek (cat. #26032). USEPA Method	GC (TQ8030 Shimadzu)	35 m × 0.25 mm x 0.5 μm. Restek Rxi 5Sil MS	MS (TQ8030 (Shimadzu)	1.2–9.0 ng/L	NR	<20	70–130	[55]
Tap & River water	DMA, EMA, DEA, DPA, TMA, DMAI. DMAPI.	No preconcentration steps. Filtered through (0.22 mm)	UFLC (Shimadzu LC-20ADXR)	ThecolumnwasaPhenomenex Polar-RPC-18 column (150× 2.0 mmI.D,4 mm particlesize,	TMS (A 4000Q, AB SCIEX, Concord)	0.02–1 μg/L	NR	<13.8	88.5–116	[56]
WTP	NDMA, NMEA, NPyr, NDEA, NPip, NMor, NDPA, NDBA, NDPhA, NNN, NAT, NAB, NNK, NNAL	SPE Oasis HLB cartridge/SPE absorbents LiChrolut EN (vinyl/divinyl polymer) and Ambersorb 572 (activated carbon) Sigma-Aldrich.	HPLC (Agilent 1100)	Kinetex C8 column (100 × 3.0 mm i.d., 2.6 μm; Phenomenex)	MS/MS	0.01–2.7 ng/L	0.03–8.8 ng/L	NR	53–93	[21]
Drinking Water	d6-NDMA, NDMA, NMEA, NDEA, NDPA, NPYR NMOR, NPIP, NDBA, NDPhA	SPE (CCC) (CNW Technologies) USEPA Method 521	GC Agilent 6890 N	DB-35MS was used (35% diphenyl/65% dimethyl polysiloxane)	Agilent QMSS 5975	NR	1.5–4.9 ng/L	NR	65–122	[18]
Drinking Water	NMEA, NDEA, NDMA, NDPA, NPyr, NPip, NDBA, NDMA-d6	SPME (57330-U, Supelco)	GC Agilent 7890	Agilent DB-624 column (30 m_ 0.25 mm I. D. 1.4 mm)	Agilent MS 5975.	0.12–0.79 ng/L),	0.1–0.8 ng/L	<10	77–114	[57]
Finished/tap/source water	NDMA, NDEA, NMOR, NPIP, NDBA	SPE (CCC) (Auto Trace 280, Dionex, Corp) CNW Technologie) EPA 521 method	UPLC (Agilent 1100)	A C8 (*2*) Capillary column (150 × 0.32 mm i.d., 5 *í*m)	MS/MS (API 4000 QTrap Applied Biosystems/MDS Sciex)	0.1–1 ng/L	NR	1.4–5.1	64–116	[16]
Wastewater	NDMA, NMEA, NDEA, NDBA, NPIP, NPYR, NMOR, THEOB, XAN, THEOP	SPE Strata-X polymeric (100 mg/6 mL) Phenomenex/SPE (CCC) (200 mg/6 mL), Supelco	GC	30 m × 0.25 mm i.d. × 0.25 _m SLB-IL111 (Supelco).	MS/MS A Brucker 320-MS.	<30.6 ng/L	<48.6 ng/L	<10	NR	[58]
Drinking/MilliQ water	NDMA Nmor Npyr NMEA NDEA Npip NDPA NDBA NDPhA	SPE	UPLC (Acquity HSS T3)	50 mm × 2.1 mm, × 1.8 μm particle size.	MS Waters (Micromass Quattro Premier XE)	<0.9 ng/L	NR	NR	70–90	[2]
Potable Water	NDMA, NDMA-d6 Toluene-d8	SPE (Sep-Pak AC-2 cartridge)	GC Varian 450	30 m × 0.25 mm × 1.0 lm	MS Varian 300	<2 ng/L	NR	<11.1	70–120	[59]
Drinking Water	NMEA, NDPA, NDEA, NDBA, NMor, NPip, NPyr.	LLE (with dichloromethane)	GC Agilent 6890	40 × 0.18 mm ID and 1 μm (RTX-VMS)	MS Agilent 5973	0.4–2.0 ng/L	10 ng/L	<19	95	[60]
Sewage	NDMA, NMOR, NPYR	SPE	HPLC Agilent HP1100 /GC QP2010 Shimadzu	NR	MS/MS (Acquity TQD)	NR	5.0–25 ng/L /1.0 ng/L	NR	NR	[61]
Bio solid	NDMA, NMEA, NDPA NDBA NPYR, NPIP, NDPhA	LLE (2 mL dichloromethane per g of biosolids)	HPLC (Shimadzu)	130 Å, 3.5 μm, 4.6 × 150 mm.	MS/MS API 4000 (Applied Biosystems)	0.06–5.7 ng/g	NR	NR	90–126	[62]
Meat Products (Pork Sausage)	NDMA, NMEA, NDEA, NPYR, NDPA, NPIP, NDBA	D-l-SPE	GC Varian 450	30 m × 0.25 mm × 1.0 l, DB5-MS.	MS (Varian 2200	0.01–0.12 (ng/g)	0.03–0.36 (ng/g)	<10	74–105	[51]
Deionized Water Water	NDMA, DMTA	SPME (PDMS/DVB, 65 μm, Supelco)	GC Thermo TRACE.	30 m × 0.25 mm 0.25 μm, (ZB-5ms Zebron, Phenomenex).	MS (Thermo TRACE DSQ II).	0.3–0.6 μM	2.5–16 Μm	0.2–0.8	NR	[63]
Cosmetic	NDMA, NMEA, NDEA, NPYR, NDPA, NPIP, NDBA	HS-SPME (splitless liner 0.75 mm i.d. Agilent)	GC Agilent 7890B	30 mm × 0.25 μm I.D × 0.25 μm, DB-WAX Agilent (Palo Alto)	MS Agilent 7693	0.46–36.54 ng/g	NR	<20	79	[8]

N-nitrosonornicotine NNN, N-nitrosoanatabine NAT N-nitrosoanabasine NAB, 4-(methylnitrosamino)-1-(3-pyridyl)-1-butanone NNK, 4-(methylnitrosamino)-1-(3-pyridyl)-1-butanol NNAL, Caffeine CAF; Theophylline THEOP, 1,7-dimethylxanthine XAN, Theobromine THEOB. Coconut charcoal cartridge (CCC). Not Registered (NR), Solid Phase Extraction (SPE), Secondary (2^o^), Effluent (E), Solid-phase micro extraction (SPME), high-performance liquid chromatography (HPLC), ultra-fast liquid chromatography–tandem mass spectrometry(UFLC-MS/MS), Trap mass spectrometer (TMS), Dimethylamine (DMA),Ethylmethylamine (EMA), Diethylamine (DEA), Dipropylamine (DPA), Trimethylamine (TMA), 3-(Dimethylaminomethyl)indole (DMAI), 4-Dimethylami- noantipyrine (DMAP), Electrospray Ionization (ESI), Tandem Quad (TQD) MS Technology, Water treatment plant (WTP). Qudrupole mass selective spectrometer (QMSS), (NDMA: N-nitrosodimethylamine, International Sorbent Technology (IST), Temperature: temp. Dispersive micro solid-phase extraction (D-l-SPE), Deuterated toluene (toluene-d8), Liquid-liquid extraction (LLE), Head space solid-phase micro extraction (HS-SPME).

Till date, there is a paucity of data on metal-nitrosamine complexes concentration in environmental water samples. Therefore, this review focuses on the reactions of NO functional group in NAms with certain transition metals and the applications of chromatographic techniques to the analysis of NAms and their metal complexes in the environmental waters.

## 2 Reactions of nitrosamines with metals

**Figure 1:**
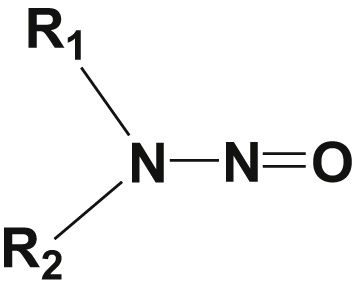
General structure of N-nitrosamines.

**Figure 2:**
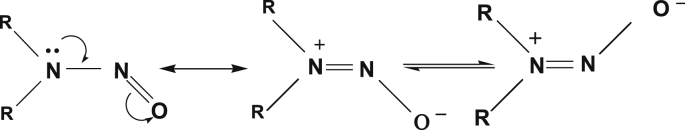
Resonance structure of N-nitrosamines.

**Figure 3:**
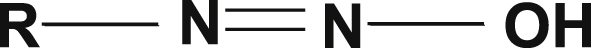
Tautomeric N-nitrosamines.

The general, resonance and tautomeric structures of N-nitrosamines are shown in Figures 1, 2, and 3 as to describe their possible reactions. Nitrosamines contain a nitrosyl group (NO) (Figure 1), the nitrogen contains five electrons in the outermost shell (valence electrons); two electrons are used to form double bonds with oxygen while three electrons left behind stay on nitrogen as one and lone pair of electrons [27, 28, 29]. Because of these features NO ligand forms structural bonding and complexes [27, 28].

### 2.1 Metallic nitrosyl bond (M-NO)

**Figure 4:**
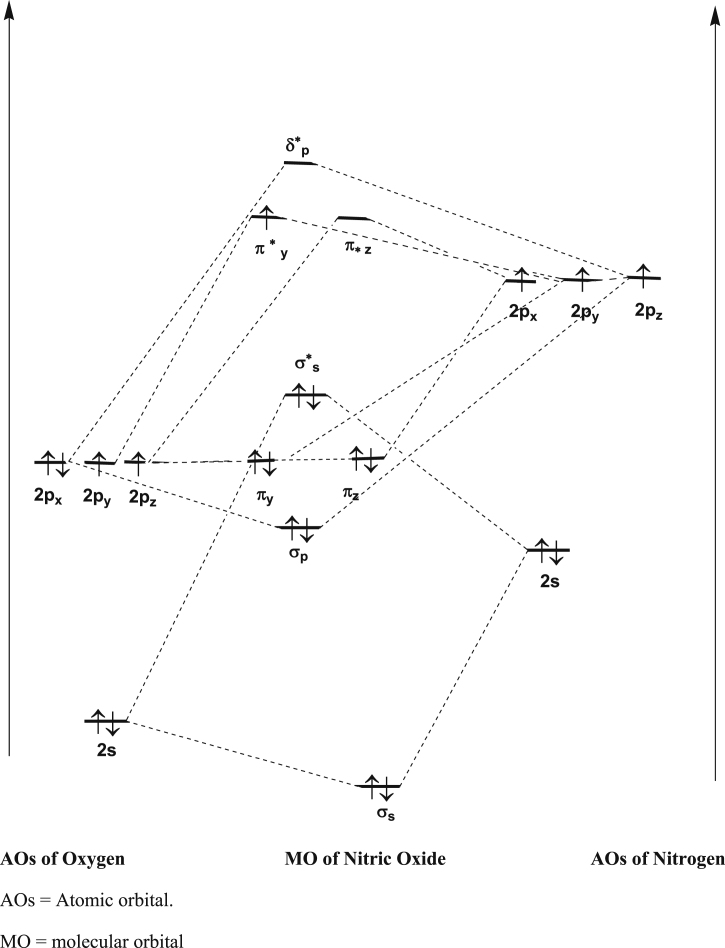
Molecular orbital (MO) pattern of nitric oxide molecule.

The general, resonance and tautomerization of N-nitrosamines are shown in Figures 1, 2, and 3. Also, for better understanding of metal-nitrosyls bond, the molecular orbital (MO) pattern of nitric oxide molecule is presented in Figure 4.

**Figure 5:**
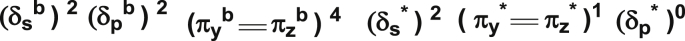
The eleven electrons used in molecular orbital bond.

The 6 and 5 electrons in the outermost shell of Oxygen and Nitrogen, respectively are used for bonding. The 11 electrons used in the formation of molecular orbital bond in NO are presented in the following order (Figure 5).

The metallic nitrosyl are as follows [27].

**Figure 6:**
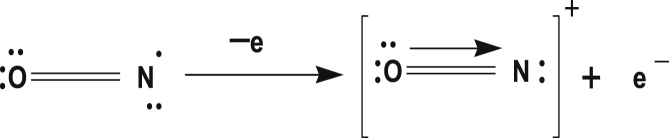
Bond formation between oxygen and nitrogen.

NO gives out an electron for formation of nitrosyl cation and oxygen releases a lone pair electron to nitrogen resulting to bond formation between oxygen and nitrogen (Figure 6).

**Figure 7:**
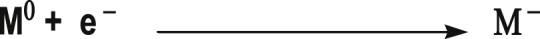
Change in the oxidation state of metal atom (M).

The unpaired electron **(π**_y_* == π_z_*) ^**1**^ received by the metal atom (M) changed its oxidation state from 0 to -1 (Figure 7) [27].

**Figure 8:**
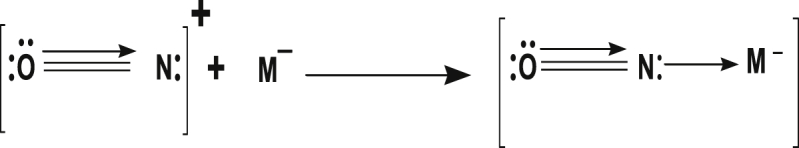
Coordinate covalent bond formation.

The Nitrogen in the **NO**^**+**^ (nitrosonium ion or nitrosyl) give out a lone pair of electron to M^−-^ for coordination (Figure 8).

Nitrosyl complexes contain NO^+^ ligand. NO^+^ has three electrons, one is donated to metal ion before the donation of a lone pair of electron for coordinate covalent bond formation (Figure 8) [27, 30, 31].

### 2.2 Formation of dative sigma (ϭ) bond

**Figure 9:**
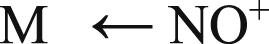
Movement of electron for the dative sigma bond formation.

**Figure 10:**
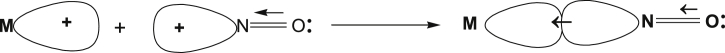
Formation of dative sigma bond.

The dative sigma (ϭ) bond is formed due to the overlapped of the empty hybrid orbital (d, s, and p – orbital) of metal atom with the filled hybrid orbital (HOMO) of the nitrogen atom of the (NO^+^) ion Figures 9 and 10 [27].

### 2.3 π-Bond formation

The nitrogen in the NO^+^ as electron acceptor leads to formation of pie (π) bond.

**Figure 11:**
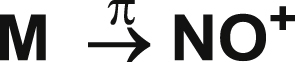
Movement of π electron for the Formation of pie bond.

**Figure 12:**
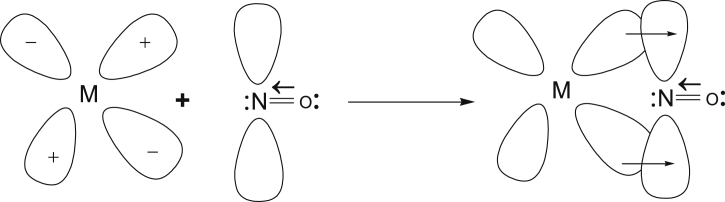
Formation Pie bond.

The hybridized dπ or dpπ of the metal atom overlapped with empty orbital NO^+^ to form π-bond as shown in Figures 11 and 12 [27,32].

**Figure 13:**
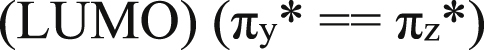
The lowest unoccupied molecular orbitals.

The lowest unoccupied molecular orbitals received electrons from the filled metal orbitals leading to formation of π-bond (Figure 13) [27,[32], [33], [34]].

**Figure 14:**
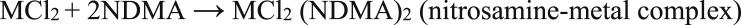
Nitrosamine-metal complex.

**Figure 15:**
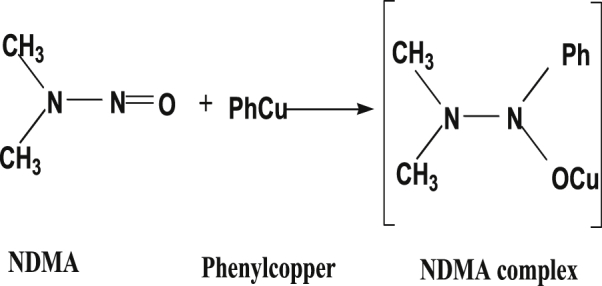
Nitrosamine-copper complex formation.

Therefore, there is the possibility of NO ligand present in NDMA forming complexes in Figures 14 and 15.

For instance, addition of NO ligand to metals lead to complexes reaction of NDMA with phenylcopper gives NDMA complex (Figure 15) [36, 37].

MCl_2_ = Chloride of transition metal [26, 30, 35].

The equations of the metal-nirosyl complexes formed in water samples have been reported by Anselme [36], Rose and Jurs [37] Kumar et al. [25] and Wang and Mitch, [26].

## 3 Application of chromatography to the analysis of nitrosamines in environmental waters

Gas chromatography (GC) coupled with various detectors have been employed for the analysis of nitrosamines and other emerging disinfection by–products (EDBPs) (Table 3). These include gas chromatography (GC) with electron capture detector (GC-ECD), nitrogen–phosphorus detector (GC/NPD), thermal energy analyzer (GC/TEA), GC with flame ionization detector (FID), GC coupled with mass spectrometry (GC-MS) [38, 39, 40, 41, 42].

The GC-ECD operates based on the ability of the organic compound to capture a thermal electron and form negatively charged ions. The electron loss is proportional to the quantity of analyte in the sample [43, 44, 45]. It is mostly used for the analysis of nitroaromatic compounds, halogen-containing compounds and conjugated compounds containing weak electrophore groups that can be improved with chemical derivatization [44, 45]. ECD is highly sensitive mostly employed for trace analysis. It has the capability to detect analyte at pictogram (10^−13^) levels [42, 46, 47, 48]. Similarly, Chienthavorn et al. [41] quantified four nitrosamines (NDEA, NPYR, NPIP, NMOR) with GC-FID. However, GC-ECD and FID have no library data base for the confirmation of the analyte, while GC-MS possesses. In addition, a good precision and linearity have been reported in the analysis of nitrosamines with GC-MS [49, 50, 51, 52, 53].

GC-MS technique explains the abundance of molecular composition and the amount of analyte in the sample in relation to the peak area [45, 64]. Its sensitivity is based on the mass of analyte received at the detector [64, 65]. Because of sensitivity and selectivity, it is used in the selected ion monitoring (SIM) mode for the analysis of thermally stable, semi-volatile, less polar and low molecular weight nitrosamines [23, 45, 51, 65, 66]. The study of nitrosamine has been narrowed to the US EPA eight semi-volatile nitrosamine analysis (Method 521) and less attention is given to the non-volatile nitrosamines. The non-volatile NAms (N-nornicotine, N- piperazine) and labile NDPhA are not amenable to GC-MS methods because they are highly polar and thermally unstable respectively [60, 67, 68, 69]. Also, gas chromatography–tandem mass spectrometry (GC/MS/MS) could gives better sensitivity and selectivity than GC-MS, but cannot be used for the analysis of non-volatile nitrosamines [7, 70, 71]. Analysis of nitrosamines using gas chromatography–low resolution mass spectrometry (GC/LRMS) in electrospray ionization (ESI) mode has also been applied in the analysis of nitrosamines. But its demerit is that it causes chemical interference during low molecular mass nitrosamines analysis [39, 72].

### 3.1 Choice of the columns in the gas chromatographic analysis of nitrosamines

The capacity of gas chromatographic separation column depends on the type of stationary phase and its polarity and the amount of the packing material used (Table 3). This increases the efficiency of the column [49, 58, 73]. A good separation is attained by the distribution of the analytes (solute) on the stationary phase (composition of the adsorbent) and a gas phase that penetrates the stationary phase [74]. Low molecular mass gases are used as mobile phase for the adequate transportation of the solute through the column [74].

**Figure 16:**
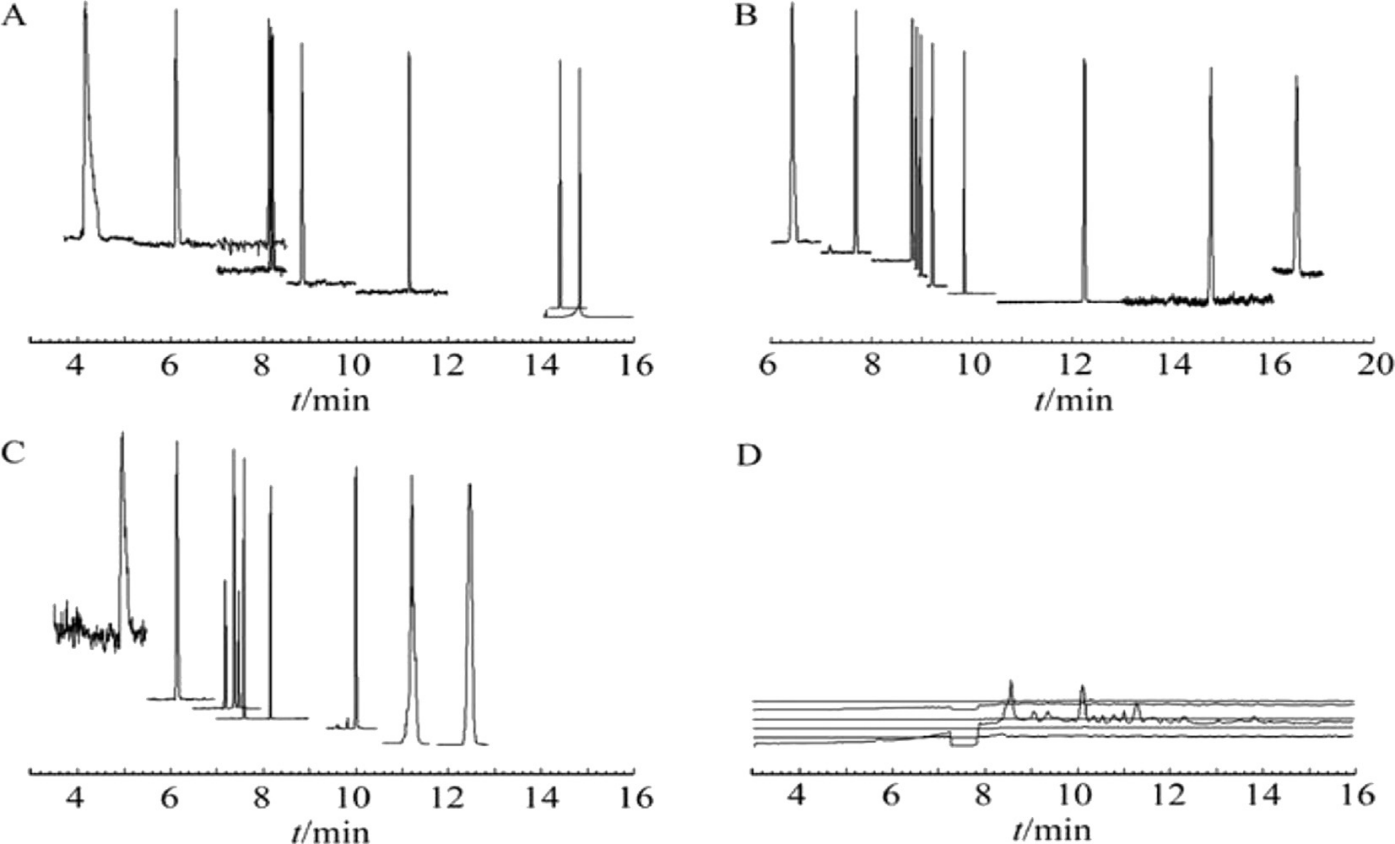
**Separation of ten volatile nitrosamines using four different gas chromatographic columns.** A, HP-5MS (30 m × 0.25 mm × 0.25 μm) column; B, DB-624 (30 m × 0.25 mm × 1.40 μm) column; C, HP-1701 (30 m × 0.53 mm × 1.0 μm) column; D, HP-INNOWax (30 m × 0.25 mm × 0.25 μm) column (Qiang et al., 2011). It has been published before in Chinese Journal Analytical Chemistry and permission to reproduce the figure has been granted.

**Figure 17:**
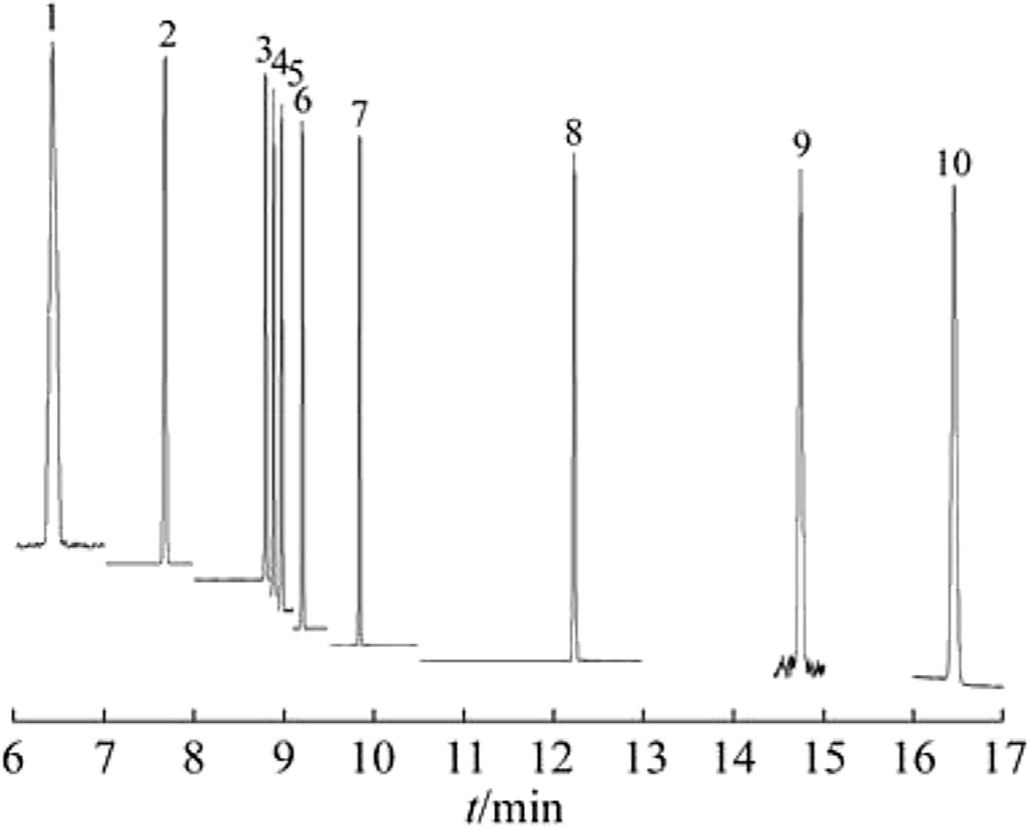
**The final separation of the ten volatile nitrosamines** DB-624 (30 m × 0.25 mm × 1.40 μm) column. 1. NMDA 2. *NDEA 3. NDPA 4. NMOR 5. NPYR 6. NPIP 7. NDBA 8.* NDPHA 9. NDCHA 10. *NDBZA* [49]. It has been published before in Chinese Journal Analytical Chemistry and permission to reproduce the figure has been granted.

A more polar stationary phase retains polar analytes better than less polar solute while a non-polar stationary phase retains any member of homologous series [49, 58]. Different types of column ranging from non-polar (HP-5MS, 5% phenyl-95% dimethylpolysiloxane; DB-5ms 5% Phenyl 95% dimethylpolysiloxane), mid-polar (DB-624, 6% cyanopropyl phenyl-94% dimethylpolysiloxane; DB-1701, 14% cyanopropylphenyl-86% dimethyl polysiloxane) and polar polar column (HP-INNOWax, Polyethylene glycol) have been reported for the analysis of nitrosamines Qiang et al. [49] used column HP-5MS, DB-624, DB-1701 and HP-INNOWax for the analysis of ten nitrosamines and they reported the best peak separation with DB-624 column. The chromatograms reported were shown in Figure 16(A–D) and Figure 17.

In the non-polar column HP-5MS small background level or low signal to noise ratio, better peak resolution at the beginning and end but poor separation of NDPA, NMOR, NPYR, NPIP at the mid of the chromatogram (Figure 16A). In Figure 16B there was a negligible background noise and all peaks were well separated throughout the chromatogram. High signal to noise ratio at the beginning, analytes were well separated at the end but fairly separated at the mid of the chromatogram (Figure 16C). Figure 16D depicted poor sensitivity and an unreproducible chromatogram could be due to absence of cyanopropyl phenyl and dimethylpolysiloxane.

Similarly, a sharp peak separation with DB-624 column was also reported as shown in Figure 17 [57, 70, 75, 76]. However, there is no published literature on column with 100% dimethylpolysiloxane which could properly yield a better separation.

## 4 Conclusions

This survey could not find a study specifically dedicated to quantitative determination of metal-complexed nitrosamines. The reason is unknown to us. An overview of the analytical methods has shown that different methods exist for analysis of nitrosamines. However, in our view, the existing reports on total nitrosamine concentration in waters may have therefore been severely underestimated. Gas chromatography with so called mid-polar column made of cyanopropyl phenyl and dimethylpolysiloxane in the ratio 1: 16 (DB-624) and linked mass spectrometer detector is one technique adaptable to the determination of metal-complexed nitrosamines in waters in view of its reproducibility and sensitivity. Attention should focus on the high molecular weight, emerging unregulated and highly toxic nitrosamines as well as metal-nitrosyl complexes occurrence in water. Also, in order to obtain a high quality chromatogram, the chemical composition of the stationary phase used in the column should be improved.

## Declarations

### Author contribution statement

All authors listed have significantly contributed to the development and the writing of this article.

### Funding statement

This research did not receive any specific grant from funding agencies in the public, commercial, or not-for-profit sectors.

### Competing interest statement

The authors declare no conflict of interest.

### Additional information

No additional information is available for this paper.
